# Particle Size in Secondary Particle and Magnetic Response for Carrageenan Magnetic Hydrogels

**DOI:** 10.3390/gels5030039

**Published:** 2019-08-10

**Authors:** Junko Ikeda, Daichi Takahashi, Mayuko Watanabe, Mika Kawai, Tetsu Mitsumata

**Affiliations:** 1 Graduate School of Science and Technology, Niigata University, Niigata 950-2181, Japan; 2 Nihon Rufuto Corporation, Tokyo 110-015, Japan; 3 ALCA, Japan Science and Technology Agency, Tokyo 102-0076, Japan

**Keywords:** stimuli-responsive material, magnetic gel, viscoelastic property, magnetorheology, carrageenan

## Abstract

The relation between the number of magnetic particles and the change in storage modulus induced by a magnetic field was investigated for weak hydrogels containing carbonyl iron, iron oxide, and barium ferrite particles with different diameters in primary particles while maintaining the magnetization of magnetic particles. The change in storage modulus exhibited a power dependency against the number of magnetic particles, which was nearly independent of the magnetic particles. The change in storage modulus was successfully scaled by the reduced number of magnetic particles using the diameter of secondary particles. Microphotographs revealed that iron oxide and barium ferrite particles form aggregations while carbonyl iron particles are well dispersed in carrageenan gels. The diameter of secondary particles determined by a relation between the change in storage modulus and the reduced number of magnetic particles showed similar values with those observed in microphotographs.

## 1. Introduction

Magnetic hydrogels made of polysaccharides have been widely investigated thus far, and many functions and applications have been reported, such as recoverable adsorbent or drug delivery. This demonstrates a unique property or stimuli-responsive behavior that is not observed in magnetic hydrogels made of synthetic polymer. Most of these unique properties originate from the relatively strong interaction between magnetic particles and polysaccharides. Polysaccharides self-organize to adhere on the surface of magnetic particles in pure water. For example, Samoilova et al. developed an excellent and facile method to prepare magnetic composites in which magnetite particles are densely covered with polysaccharides [1,2], showing effective adsorbents for specific lectins and enzyme. A novel adsorbent of magnetic gel beads consisting of carboxymethyl chitosan, sodium alginate, graphene oxide, and iron oxide is utilized for the adsorption of metal ions from wastewater [3].

Carrageenan an appropriate polysaccharide for synthesizing magnetic hydrogels because of its high zeta potential due to the presence of anionic groups on its chains. A carrageenan hydrogel with Fe_3_O_4_ nanoparticles demonstrated applicability in controlled drug release [4]. Micron-sized carrageenan hydrogel particles exhibited increased amounts of drug absorption and release [5]. Magnetic oxidized multiwalled carbon nanotube/carrageenan/Fe_3_O_4_ nanocomposites were investigated as an adsorbent for the removal of methylene blue from aqueous solution [6]. In particular, magnetic nanoparticles made of polysaccharides are actively investigated for their use in biological systems such as in pharmacy or hyperthermia technology [7,8,9,10]. Thus, polysaccharide that is obtained from a biomass has many advantages not only in its safety in biological systems, biocompatible or biodegradable properties, but also many functionalities.

Carrageenan magnetic hydrogels or other polysaccharide magnetic hydrogels demonstrate various unique phenomena on the magnetorheological effect (MR effect) [11,12,13,14], e.g., the storage modulus for carrageenan or agar magnetic hydrogels containing carbonyl iron particles showed a high MR effect even in a dense polymer network [15]. Possible reasons for this include the ability of polysaccharide networks to transfer stress due to the indirect contact between magnetic particles via a polysaccharide network; otherwise, magnetic particles can move easily even in the dense polymer network because of bonding and detaching of hydrogen bonds. As mentioned above, the adhesion force acting between carbonyl iron particles and polysaccharide chains would be a key factor for the high MR effect. However, details of the mechanism—unique to the MR effect of polysaccharide magnetic hydrogels—have not yet been clearly described.

To characterize the high MR effect observed for carrageenan/carbonyl iron magnetic hydrogels, the effect of magnetic particles on the MR effect was investigated in this study using carbonyl iron, iron oxide, and barium ferrite particles. Carbonyl iron has good dispersibility while iron oxide and barium ferrite form agglomerates in carrageenan aqueous solutions or gels. The effect of aggregation or agglomeration of magnetic particles on the MR effect is studied using scaling analysis, which is a plot of the change in storage modulus vs. the number of magnetic particles. The magnetization is different for these magnetic particles. To eliminate the difference in magnetization for these magnetic particles, we carried out the rheological measurement at a certain magnetic field so that the particles have the same magnetization (=40 emu/g). The increment in the storage modulus due to magnetic fields should be proportional to the number density of chains of magnetic particles that is a function of the number of magnetic particles. Therefore, the increment in the storage modulus should be scaled by the number of magnetic particles irrespective of the kind of magnetic particle. In this study, we investigated the effect of magnetic particles on the change in storage modulus for carrageenan magnetic hydrogels and discovered the key parameter that decides the change in storage modulus for these magnetic gels.

## 2. Results and Discussion

**Figure 1:**
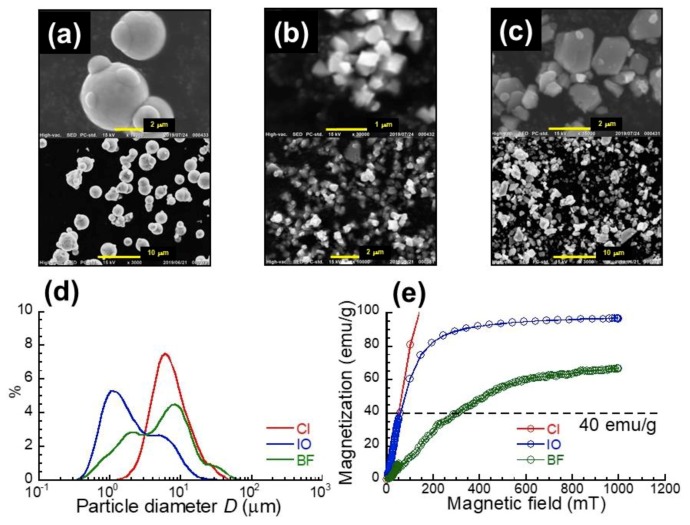
SEM photographs for (**a**) carbonyl iron, (**b**) iron oxide, and (**c**) barium ferrite particles. (**d**) Particle diameter distribution and (**e**) magnetization curves for these magnetic particles.

Figure 1a–c display the SEM photographs for carbonyl iron (CI), iron oxide (IO), and barium ferrite (BF) magnetic particles, respectively. CI particle has a spherical shape while IO and BF particles have irregular shapes. Figure 1d shows the distribution of particle diameter for CI, IO, and BF particles measured by a particle size analyzer. CI particles exhibited a single peak at 6.1 μm, while IO and BF particles showed a shoulder besides the main peak. The main peak for IO and BF particles was 1.1 and 7.8 μm, respectively. The averaged median diameter for CI, IO, and BF particles was 6.7, 1.7, and 4.8 μm, respectively. Figure 1e exhibits the magnetization curves for CI, IO, and BF particles by a magnetometer. The change in storage modulus is proportional to the square of magnetization of magnetic particles similarly to the Maxwell stress [11]. Therefore, the magnetization should be unified for these magnetic particles. When the magnetization was 40 emu/g, the magnetic field for CI, IO, and BF particles was 50, 50, and 300 mT, respectively. These magnetic field strengths were chosen in the viscoelastic measurement in order to give the same magnetization for these magnetic particles.

**Figure 2:**
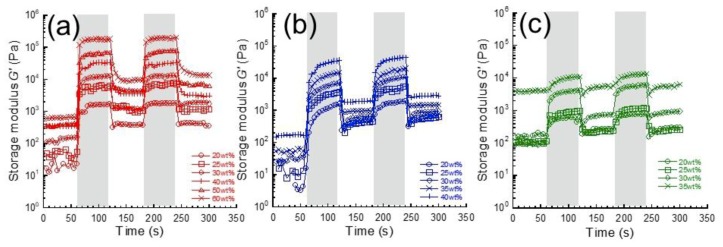
Magnetic response of storage modulus at *γ* = 10^−4^ and 1 Hz for carrageenan magnetic hydrogels with (**a**) carbonyl iron, (**b**) iron oxide, and (**c**) barium ferrite particles with various concentrations of magnetic particles. Magnetic fields were applied in the shadowed area.

Figure 2 depicts the magnetic response of storage modulus in the linear viscoelastic regime for carrageenan magnetic hydrogels containing CI, IO, and BF particles with various concentrations of magnetic particles. The storage modulus before the first application of the magnetic field was distributed from 10 Pa to 1 kPa, suggesting these are weakly crosslinked gels. All magnetic hydrogels exhibited a change in storage modulus in response to the pulsatile magnetic field. The increase in the storage modulus is due to the chain formation of magnetic particles by magnetic fields. In fact, we successfully observed the chain structure of magnetic particles in a magnetic elastomer using computed tomography at high resolution [16].

**Figure 3:**
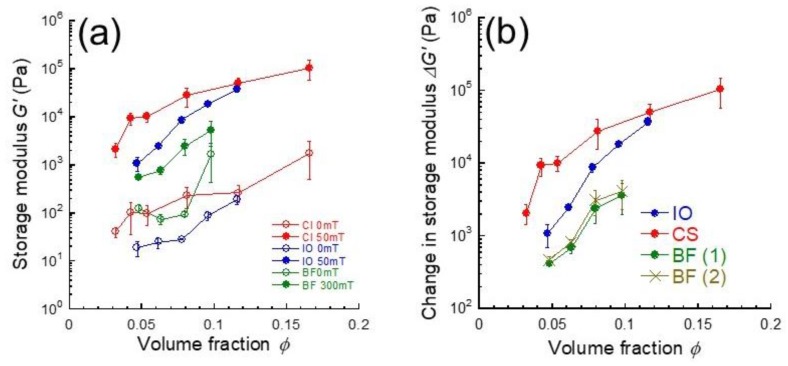
Volume fraction dependence of (**a**) off-field and on-field storage modulus and (**b**) change in storage modulus for carrageenan magnetic hydrogels with carbonyl iron, iron oxide, and barium ferrite particles (*γ* = 10^−4^, *f* = 1 Hz). BF (1) and BF (2) are the change in storage modulus by the first application and second application of the magnetic field, respectively.

Figure 3a demonstrates the relationship between the storage modulus and volume fraction of magnetic particles for carrageenan magnetic hydrogels containing CI, IO, and BF particles. Both storage moduli with and without magnetic field at the beginning below 120 s in Figure 2 were used. All magnetic gels demonstrated that the storage modulus at 0 and 50 mT (or 300 mT) increased with the volume fraction of magnetic particles. The volume fraction dependence of storage modulus for magnetic fluids of spherical particles has been investigated by Martin and Anderson, de Vicente, or Ginder. All models showed the storage modulus is proportional to the volume fraction of magnetic particles [17,18,19]. There are also reports describing about the yield stress that it linearly increased with the volume fraction of magnetic particles at *Φ* < 0.2 for magnetic fluids of both carbonyl iron and Fe_3_O_4_ particles and it increased exponentially at higher volume fractions [20]. Volkova et al. reported that the yield stress for ferrofluids containing carbonyl iron particles increases with the volume fraction [21]. In this study, the storage modulus in the presence of magnetic field showed a power dependency as *G*’ ~ *Φ^n^* at whole volume fractions, where the exponent *n* for CI, IO, and BF was determined to be 2.2, 4.0, and 3.3, respectively. Molchanov et al. was reported in the volume fraction dependence of storage modulus that there were two power-law dependences of *G*’ ~ *Φ*^1.5 ± 0.1^ at *Φ* < 0.01 and *G*’ ~ *Φ*^2.1 ± 0.1^ at *Φ* > 0.01 [22]. Similar dependencies, *G*’ ~ *Φ*^2.5 ± 0.3^ for medium fields and *G*’ ~ *Φ*^2.2±0.4^ for high fields, were observed for submicron magnetite particles suspended in silicon oil containing polyethylene at *Φ* < 0.05 [23]. In our study, only CI particle was in good agreement with these values. It might be that the exponent for aggregative particles takes high values compared to that for randomly dispersed particles.

Figure 3b shows the change in storage modulus due to the magnetic field as a function of the volume fraction of magnetic particles for carrageenan magnetic hydrogels with CI, IO, and BF particles. The change in storage modulus Δ*G*’ is calculated from Δ*G*’ = *G*’_B_ − *G*’_0_; where *G*’_B_ is the storage modulus under magnetic fields and *G*’_0_ is storage modulus at 0 mT. The magnetic response of the storage modulus for the first application of magnetic field was not the same as that for the second application. Accordingly, the change in storage modulus should be evaluated using both the first and second applications of magnetic field. The both results are shown in the figure. It was clearly observed from Figure 3b that the Δ*G*’ at a certain volume fraction depends on the kind of magnetic particles even though the magnetization is completely the same for these particles. There was no difference in the change in storage modulus between the first and second applications of the magnetic field. If the increment in storage modulus is simply due to the connection among magnetic particles, then Δ*G*’ should be scaled by the volume fraction of magnetic particles independently of the magnetic particles. This strongly indicates that the diameter for magnetic particles in carrageenan hydrogel is different from that in primary particles. Otherwise, the large increase in the storage modulus for CI particles may be caused by a special mechanism, e.g., stress transfer via the polysaccharide network cooperating with CI particles, or structure formation into body-centered cubic under magnetic fields [24]. The information relating to the chain structure obtained from simulations, e.g. mean chain length, number of particles in a chain [25], would be helpful to elucidate the large Δ*G*’.

**Figure 4:**
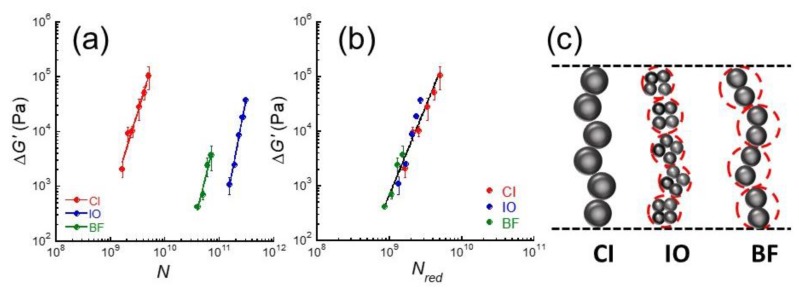
Change in storage modulus due to magnetic fields as a function of (**a**) the number of magnetic particles, (**b**) the reduced number of magnetic particles for carrageenan magnetic hydrogels with carbonyl iron, iron oxide, and barium ferrite particles (*γ* = 10^−4^, *f* = 1 Hz). (**c**) Schematic illustration for the chain structure of magnetic particles; red circles represent the secondary particles.

Figure 4a shows the change in storage modulus due to the magnetic field as a function of the number of magnetic particles for carrageenan magnetic hydrogels with CI, IO, and BF particles. The number of magnetic particles *N* was calculated from the following equation:

(1) $$N=\frac{v_{tot}}{\frac{4}{3}\pi {\left(\frac{D_{p}}{2}\right)}^{3}}$$

Here, *v_tot_* is the total volume of magnetic particles in feed, *D*_p_ is the diameter of magnetic particle in primary particles determined by a particle size analyzer. The slope of the lines for CI, IO, and BF was determined to be *N*^3.3^, *N*^5.2^, and *N*^4.1^, respectively, using least-squares fitting. Similar values in the exponent for these particles means that the Δ*G*’ can be roughly scaled by the number of magnetic particles independently of the kind of magnetic particles. Hence, three lines in Figure 4a are considered to be one line by shifting two lines for IO and BF particles toward the CI line along *N*-axis. 

Figure 4b shows the change in storage modulus due to the magnetic fields as a function of the reduced number of magnetic particles for carrageenan magnetic hydrogels with CI, IO, and BF particles. The reduced number of magnetic particles *N_red_* was also calculated from Equation (1) by substituting the diameter of primary particle *D*_p_ into the diameter of secondary particle *D*_s_. The Δ*G*’ was successfully scaled as Δ*G*’ ~ *N_red_*^3.2^ with a correlation coefficient of 0.971, independently of the magnetic particles when the diameters of secondary particles were used. Although CI particles make aggregations consisting of few particles, we hypothesized that the diameter of CI particle equals to the median diameter of primary particle (=6.7 μm). This means that the line of CI was not shifted in Figure 4a. Thus, we obtained the diameter of secondary particles for IO and BF particles as 8.4 and 9.8 μm, respectively, which corresponds to the relative diameter of secondary particles of 1.3 and 1.5 (Figure 4c), respectively, when the CI particle was regarded as a standard. As seen in Figure 4b, the change in storage modulus was scaled by the reduced number of magnetic particles. Therefore, we concluded the dominant parameter of the amplitude of MR effect as the reduced number of magnetic particles that is equal to the diameter of secondary particles. Yang et al. reported that the field-induced aggregation of iron particles can be accelerated by increasing the strength of field [26]. It would be interesting to clarify the magnetic field dependence on the diameter of secondary particles. As described above, we observed the chain structure of magnetic particles by computed tomography [16]. Chain structures consisting of IO or BF secondary particles will be reported in a subsequent paper. 

**Figure 5:**
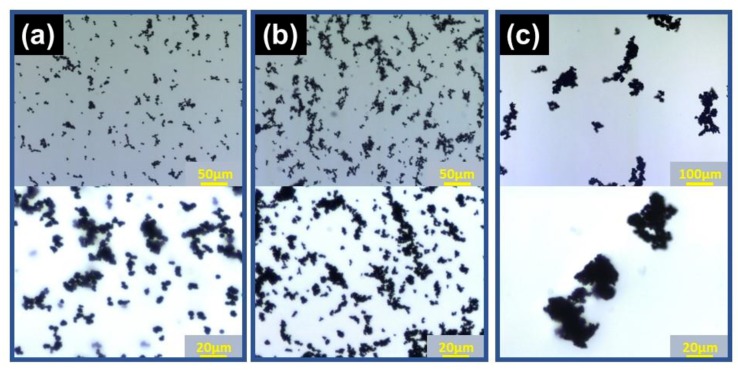
Microphotographs for magnetic particles in carrageenan hydrogels: (**a**) carbonyl iron, (**b**) iron oxide, and (**c**) barium ferrite particles (*Φ* = 5 × 10^−3^, carrageenan concentration: 0.8 wt %).

Figure 5 shows the microphotographs for carrageenan magnetic hydrogels with CI, IO, and BF particles. Image analysis was carried out for microphotographs and the dimension of secondary particles was evaluated. The averaged diameter for CI particles was determined to be 5.1 μm, which is close to the median diameter of primary particles (=6.7 μm). On the other hand, the diameter of IO and BF particles was determined to be 7.4 and 39.1 μm, respectively, which are 4- or 8-fold larger than the median diameter of primary particles (1.7 and 4.8 μm). The diameter of secondary particles for IO and BF particles with respect to CI particle was 1.5 and 7.7, respectively. The value for IO coincided with that obtained from the relation of Δ*G*’ and *N_red_* as shown in Figure 4b, however a good agreement was not obtained for BF particle. It might be for aggregative particles such as BF particle that large agglomerates are formed by a compression while preparing samples for microscope observation. It was reported that the improvements for the dispersibility of magnetic particles by polysaccharides are also effective in nanoparticles [27].

## 3. Conclusions

The basic property in magnetic response of storage modulus for carrageenan gels containing magnetic particles with different sizes in primary particles was investigated to discover their features in polysaccharide magnetic hydrogels. The volume fraction dependence of the change in storage modulus was dependent on the magnetic particles, however, it exhibited a power dependency against the number of magnetic particles for all magnetic particles. The change in storage modulus was successfully scaled by the reduced number of particles taken account for the diameter of secondary particles. This strongly suggests that the diameter of secondary particles dominates the amplitude of the change in storage modulus of magnetorheological effect for weakly crosslinked hydrogels. Microphotographs revealed that iron oxide and barium ferrite particles forms aggregations while carbonyl iron particles are randomly dispersed in carrageenan gels. The diameters of secondary particles determined by the change in storage modulus nearly coincided with those observed in microphotographs. The insights obtained here can be useful for designing magnetoresponsive materials in tactile sense devices, actuators, or sensors.

## 4. Materials and Methods

### 4.1. Synthesis of Magnetic Hydrogels

The polymer matrix of magnetic hydrogels is κ-carrageenan of a polysaccharide (*M*_w_ = 857 kDa, CS-530, San-Ei Gen F.F.I., Osaka, Japan). The gelation concentration for the carrageenan aqueous solution was approximately 0.3 wt %. The aqueous solution of carrageenan without magnetic particles was viscous with a storage modulus of 12 ± 3 Pa. In addition, magnetic particles can be stably dispersed in the solution due to the adsorption of carrageenan chains on the surface of magnetic particles. Due to the electrostatic repulsion of sulfonate groups in carrageenan, magnetic particles did not accumulate in the solution for at least 6 min during the viscoelastic measurement. The magnetic particles used were carbonyl iron (CS Grade BASF SE., Ludwigshafen am Rhein, Germany), iron oxide (Fe_3_O_4_) (Wako Pure Chemical Industries. Ltd., Osaka, Japan), and barium ferrite (Sigma-Aldrich Co., St. Louis, MO, USA). The saturation magnetization for CI, IO, and BF particles was 190, 97, and 67 emu/g, respectively. The remanent magnetization was 1.6, 14, and 56 emu/g, respectively. The coercive force was 8.5, 15, and 380 mT, respectively. A pre-gel solution of the magnetic gel was prepared by mixing carrageenan aqueous solution and magnetic particles at 100 °C using a vortex mixer for approximately 1 min. The weight concentration of magnetic particles was varied typically 20–60 wt %. The magnetization curve for magnetic particles was measured using a SQUID magnetometer (MPMS, Quantum Design Inc., San Diego, CA, US) at 25 °C. The median diameter for magnetic particles was determined by a particle size analyzer (SALD-2200, Shimadzu Co. Ltd., Kyoto, Japan).

### 4.2. Rheological Measurements

The magnetic response of storage modulus for magnetic weak hydrogels were carried out using a rheometer (MCR301, Anton Paar Pty. Ltd., Graz, Austria) at 20 °C. The oscillation amplitude and the frequency were constant at 10^−4^ and 1 Hz, respectively, at all measurements. The gap between the parallel plates was kept at 0.95 mm.

### 4.3. SEM Observations

Scanning electron microscope (SEM) observation was carried out using JCM-6000 Neoscope (JEOL Ltd., Tokyo, Japan) with an accelerating voltage of 15 kV with Au coating.

### 4.4. Microscope Observations

Observations were carried out using an upright microscope (Axio Imager M1m, Carl Zeiss, Oberkochen, Germany) with transmitted light illumination at room temperature. A pre-gel solution of magnetic hydrogels containing magnetic particles with a volume fraction of 5 × 10^−3^ were prepared on a preparation glass. A droplet of the pre-gel solution heated at approximately 80 °C was dropped on a preparation glass and it was covered by a square cover glass with a size of (22 × 22) mm^2^. Nine sheets of photos were taken at three different places in the sample for three different samples. The size distribution (area) of magnetic particles was analyzed using the software Auto Measurement (Carl Zeiss, Oberkochen, Germany). The diameter of the magnetic particles of secondary particles was determined by assuming its shape as sphere, although IO and BF particles were not spherical, as can be seen from the microphotographs.
